# Dihydromyricetin protects against liver ischemia/reperfusion induced apoptosis *via* activation of FOXO3a-mediated autophagy

**DOI:** 10.18632/oncotarget.12894

**Published:** 2016-10-25

**Authors:** Yongbiao Chen, Lizhi Lv, Huifeng Pi, Weijia Qin, Jianwei Chen, Dengfang Guo, Jianyu Lin, Xiaobing Chi, Zhelong Jiang, Hejun Yang, Yi Jiang

**Affiliations:** ^1^ Department of Hepatobiliary Surgery, Fuzhou General Hospital of PLA, Fuzhou, Fujian, China; ^2^ Department of Hepatobiliary Surgery, Dongfang Hospital of Xiamen University, Fuzhou, Fujian, China; ^3^ Department of Occupational Health, Third Military Medical University, Chongqing, China; ^4^ The 517th Hospital of PLA, Xinzhou, Shanxi, China; ^5^ Department of Hepatobiliary Surgery, Fuzhou General Hospital of Fujian Medical University, Fuzhou, Fujian, China; ^6^ Department of General Surgery, Mindong Hospital of Fujian Medical University, Fuan, Fujian, China

**Keywords:** dihydromyricetin, FOXO3a, autophagy, liver ischemia/reperfusion

## Abstract

Liver ischemia and reperfusion (I/R) injury is characterized by defective liver autophagy accompanied by alterations to the endogenous defense system. Dihydromyricetin (DHM) is a natural flavonoid that demonstrates a wide range of physiological functions, and has been implicated as a regulator of autophagy. This study investigates the protective effects of DHM pretreatment on liver injury caused by ischemia/reperfusion (I/R) and elucidates the potential mechanism of DHM-mediated protection. Mice were subjected to 60 minutes of ischemia followed by 5 hours of reperfusion. DHM (100 mg/kg bw/day) or the vehicle was administered daily by gavage 7 days before ischemia and immediately before reperfusion. In this study, DHM markedly decreased serum aminotransferase activity and inhibited liver I/R -stimulated apoptosis. Moreover, DHM exerted hepatoprotective effects by upregulating mRNA levels of various essential autophagy-related genes including ATG5, ATG12, BECN1, and LC3. Autophagy inhibitor chloroquine or Atg5 knockdown blocked DHM -mediated elevation in liver function. Specifically, DHM significantly increased FOXO3a expression, and enhanced FOXO3a nuclear translocation and Ser588 phosphorylation modification. Importantly, the inhibition of FOXO3a with FOXO3a-siRNA in mice decreased DHM-induced autophagy-related genes and diminished the protective effects of DHM against liver I/R injury. In summary, these findings identify DHM as a novel hepatoprotective small molecule by elevating FOXO3a expression and nuclear translocation, stimulating autophagy-related genes and suppressing liver I/R-induced apoptosis, suggesting FOXO3a may have therapeutic value in liver cell protection in liver I/R injury.

## INTRODUCTION

Liver ischemia/reperfusion (I/R) injury, caused by blood deprivation followed by reperfusion, occurs in various clinical settings, including liver surgery, transplantation, and resuscitation from shock [1-3]. Developing protective strategies to reduce I/R injury is of paramount importance, as liver I/R injury has been implicated as a potent contributor to an increased rate of acute liver failure, graft rejection, and chronic liver dysfunction following liver transplantation.

Autophagy is a highly conservative cellular process involving the degrading and recycling of bulk cytosolic proteins and damaged organelles to maintain cellular homeostasis [4]. A growing body of evidence indicates that autophagy plays an important role in several liver diseases, including alcoholic liver disease, non-alcoholic fatty liver disease, viral hepatitis, toxin- or drug-induced liver damage, and hepatocellular carcinoma [5-7]. Autophagy also acts as a protective mechanism during hepatic I/R injury, and induction of autophagy has emerged as a new potential strategy to ameliorate liver function after I/R injury [8, 9].

Dihydromyricetin (DHM), a Rattan tea extract, is a flavonoid reported to have a broad range of health benefits, including improving insulin sensitivity, as well as anti-inflammatory, antioxidant, hepatoprotective, and anti-tumor properties [10-13]. Recent studies have suggested that the autophagy enhancement by DHM protects against type 2 diabetes. DHM exerts anti-insulin resistance effects by inducing autophagy *via* activation of the AMPK-PGC-1a-Sirt3 signaling pathway [14]. However, the mechanisms by which DHM regulates autophagy in liver I/R injury remain undefined.

The forkhead family of transcription factors participates in regulating diverse cellular functions such as apoptosis, differentiation, metabolism, proliferation, and survival [15]. In particular, FOXO3a is reported as a potent transcriptional activator responsible for induction of autophagy-related genes [16]. Recently, FOXO3a has been shown to protect against liver injury from ethanol by inducing autophagy, which indicates that autophagy may be an essential mediator of protection conferred by FOXO3a [17].

Based on these findings, we hypothesize that DHM could protect against liver I/R injury by inducing autophagy, a process that may be mediated by FOXO3a. Our results indicate that FOXO3a is required for the hepatoprotective effects of DHM, as FOXO3a inhibition abolished its observed protection in liver I/R injury *in vivo*.

## RESULTS

### DHM reduces liver I/R injury in C57BL/6 mice

**Figure 1:**
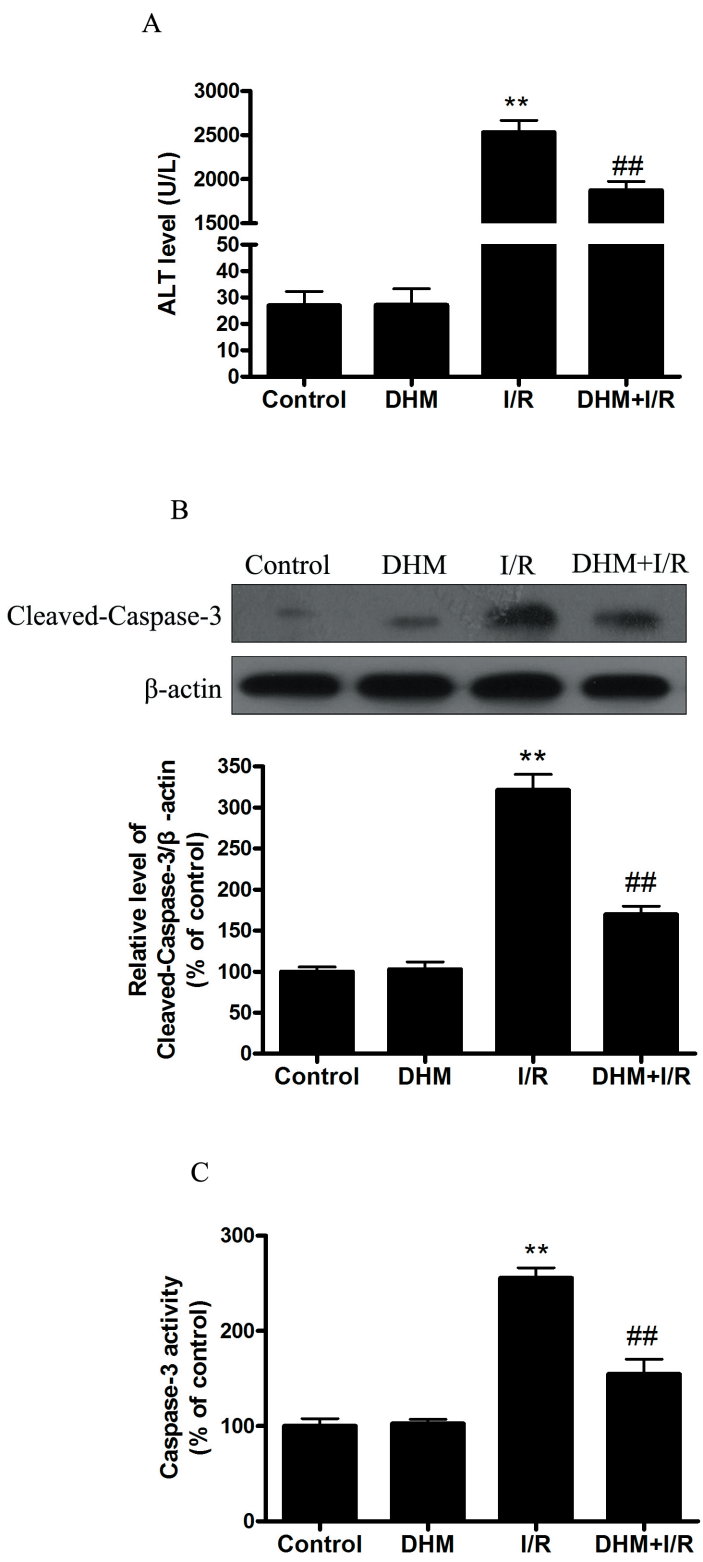
DHM reduces liver I/R injury in C57BL/6 mice **A.** ALT levels were determined using an ALT Determination Kit. **B.** Representative immunoblot of Cleaved-Caspase-3 protein levels (17 kDa) in mice liver tissue. β-actin was the internal standard for protein loading. **C.** Caspase-3 activities in mice liver samples were determined using a Caspase-3 Activity Determination Kit. The results are expressed as a percentage of control, which was set at 100 %. The values are presented as the means ± SEM, **p < 0.01 *versus* the control group, and ^##^*p* < 0.01*versus* the liver I/R group (*n* = 20).

Serum ALT activity, which is a serum marker of hepatocyte injury, was 27 ± 2.5 U/L in the vehicle-treated sham animals. The ALT levels were similar in the vehicle- and DHM-treated sham animals. However, serum ALT levels in the ischemic group 5 hours after reperfusion were approximately 90 times those observed in the sham. These increases were reduced by DHM treatment. (Figure 1A). Apoptotic hepatocytes were detected by cleaved-caspase-3 western blot. Cleaved caspase-3 was barely detectable in liver tissue obtained from the sham-operated animals. In contrast, the cleaved caspase-3 content had increased three fold at 5 hours after reperfusion. This increase was prevented by DHM treatment (Figure 1B). Moreover, results shown in Figure 1C suggest that caspase-3 activity in the vehicle- or DHM-treated sham was quite low. However, caspase-3 activity was significantly higher after 5 hours of reperfusion, which was prevented by the DHM treatment.

### DHM suppressed liver I/R injury by activation of autophagy in C57BL/6 mice

**Figure 2:**
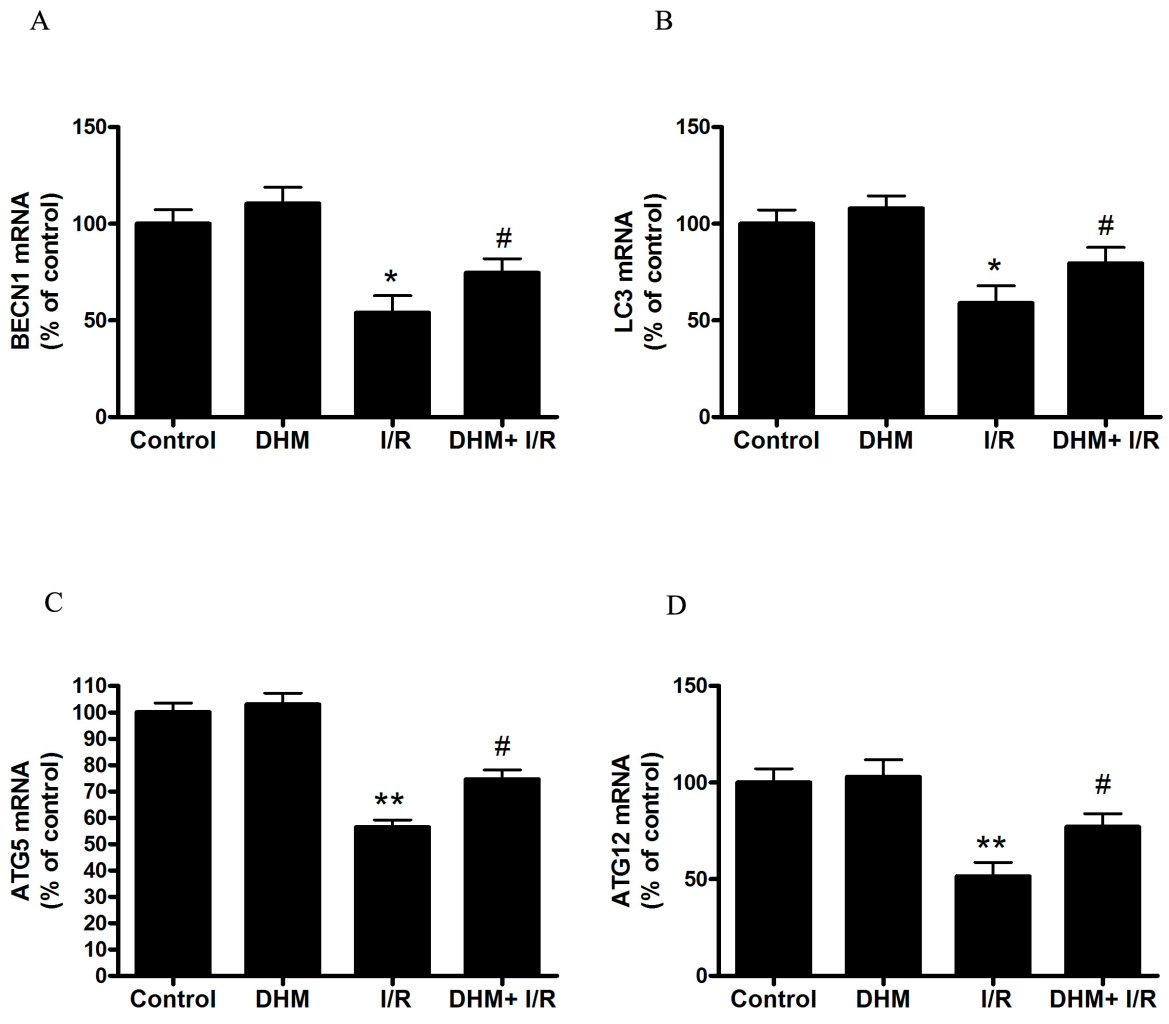
Effects of DHM on the expression of autophagy-related genes suppressed by liver I/R injury in C57BL/6 mice The mRNA level of **A.** BECN1, **B.** LC3, **C.** ATG5, and **D.** ATG12, was determined using RT-PCR as described previously. The results are expressed as a percentage of control, which was set at 100 %. The values are presented as the means ± SEM, **p < 0.01 *versus* the control group, and ^#^*p*< 0.05 *versus* the liver I/R group(*n* = 20).

**Figure 3:**
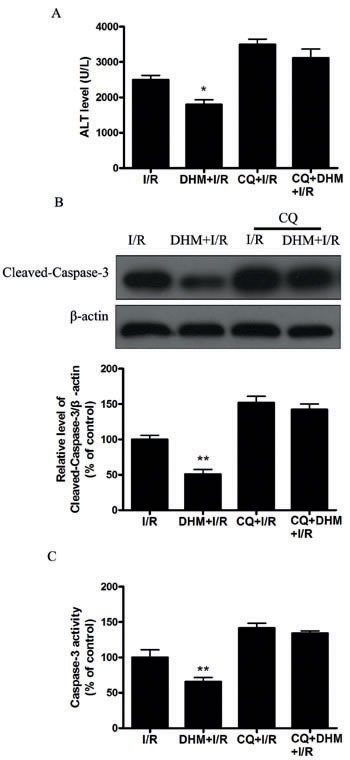
DHM decreases liver injury after I/R through induction of autophagy and abolished by CQ Mice were pretreated with CQ (60 mg/kg, i.p.) 0.5 h prior to DHM treatment. **A.** ALT levels were determined using an ALT Determination Kit. **B.** Representative immunoblot of Cleaved-Caspase-3 protein levels (17 kDa) in mice liver tissue. β-actin (42 kDa) was the internal standard for protein loading. **C.** Caspase-3 activities in mice liver samples were determined using a Caspase-3 Activity Determination Kit. The results are expressed as a percentage of control, which was set at 100 %. The values are presented as the means± SEM, *p < 0.05, **p < 0.01 *versus* the liver I/R group (*n* = 20).

**Figure 4:**
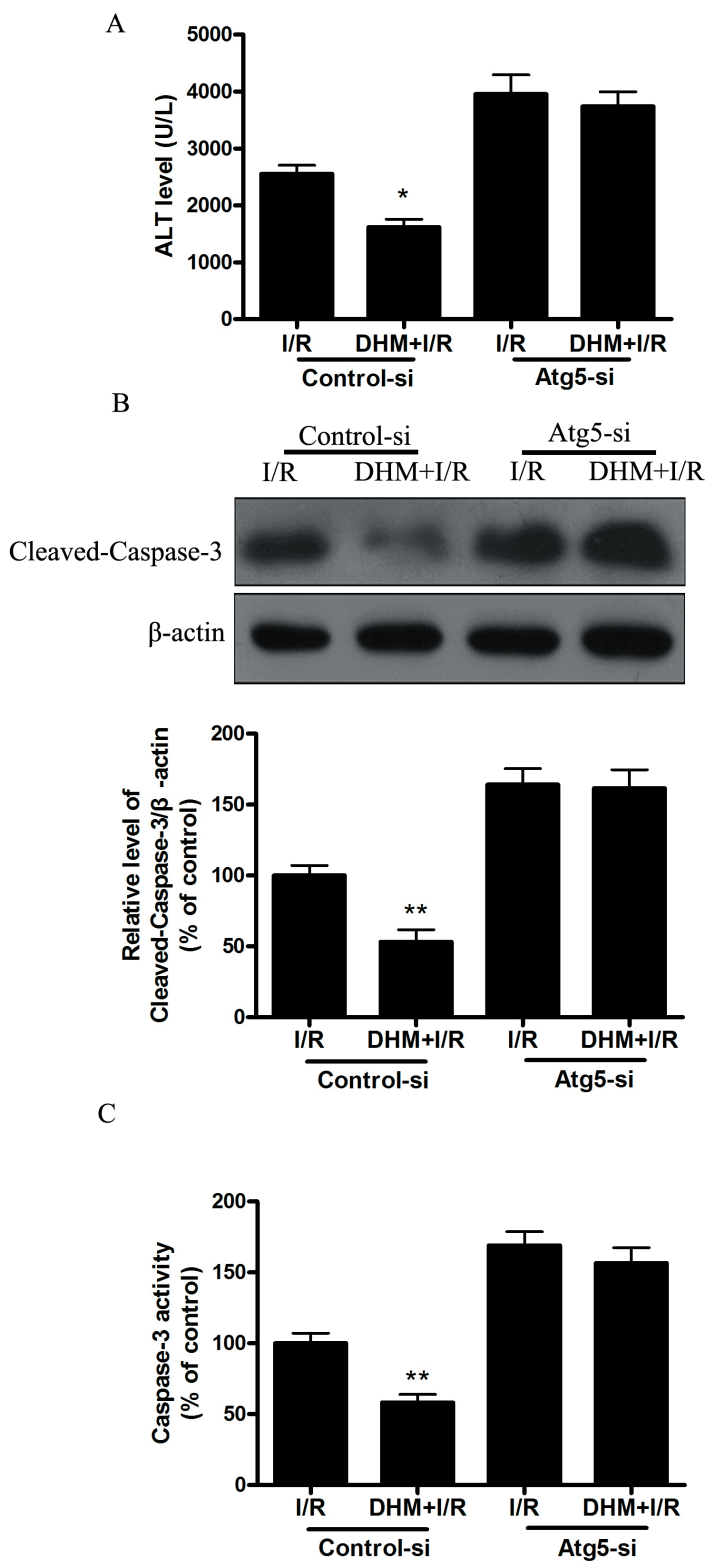
DHM decreases liver injury after I/R through induction of autophagy and abolished by Atg-5-si Mice were pretreated with Atg-5-si prior to DHM treatment. **A.** ALT levels were determined using an ALT Determination Kit. **B.** Representative immunoblot of Cleaved-Caspase-3 protein levels (17 kDa) in mice liver tissue. β-actin (42 kDa) was the internal standard for protein loading. **C.** Caspase-3 activities in mice liver samples were determined using a Caspase-3 Activity Determination Kit. The results are expressed as a percentage of control, which was set at 100 %. The values are presented as the means± SEM, *p < 0.05, **p < 0.01 *versus* the liver I/R group (*n* = 20).

Autophagy helps cells survive by conferring apoptosis resistance; inhibition of autophagy causes caspase-dependent cell death [18]. We investigated whether DHM had a protective effect on the liver I/R -induced apoptotic pathway and liver dysfunction by autophagy activation. To determine whether DHM treatment activated autophagy, we examined the mRNA levels of various essential autophagy-related genes, including ATG5, ATG12, BECN1, and LC3, involved in the regulation and initiation of the autophagic process. After hepatic I/R injury, the mRNA levels of ATG5, ATG12, BECN1, and LC3 expression significantly decreased compared with those of the control group. DHM treatment attenuated the decreases (Figure 2A-2D). To determine whether DHM operated by activating autophagy to inhibit liver I/R injury, C57BL/6 mice were pretreated with DHM for 7 days and then exposed to hepatic I/R with or without the autophagy inhibitor, chloroquine (CQ). CQ pretreatment reversed the protective effects of DHM on hepatic I/R -induced ALT production (Figure 3A). Moreover, as shown in Figures 3B and 3C, DHM-induced decreases in Cleaved-Caspase-3 expression and Caspase-3 activity were significantly attenuated by CQ in liver I/R injury. Moreover, inhibition of autophagy by Atg5 knockdown also abolished the protective effects of DHM (Figure 4A-4C).

### Autophagy-protective action of DHM is FOXO3a dependent in liver I/R injury in C57BL/6 mice

**Figure 5:**
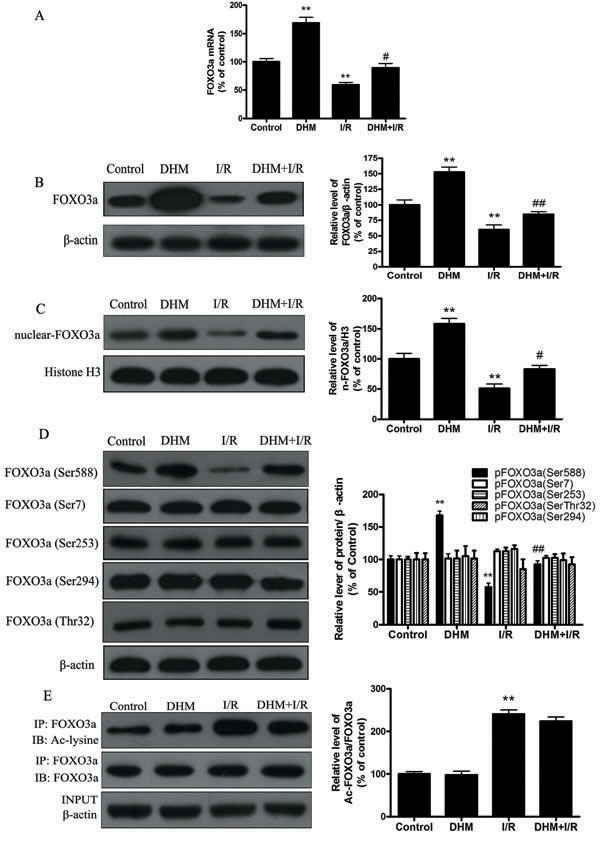
DHM significantly increases FOXO3a expression, and enhances FOXO3a nuclear translocation and phosphorylation modification after I/R **A.** The mRNA level of FOXO3a. **B.** Representative immunoblot of FOXO3a protein levels, with β-actin as an internal loading control. **C.** Representative immunoblot of nuclear FoxO3a levels, with H3 as an internal loading control. **D.** p-FOXO3a(Ser588), p-FOXO3a(Ser7), p-FOXO3a(Ser294), p-FOXO3a(Ser253) and p-FOXO3a(Thr32) protein expression levels were measured using Western blot analysis as described previously, with β-actin as an internal loading control. **E.** Acetylation of FOXO3a by immunoprecipitation with an FOXO3a antibody, followed by immunoblot analysis of anti-acetylated-lysine. A representative Western blot and the quantification of the ratio of acetylated FOXO3a to FOXO3a are shown. The results are expressed as a percentage of the control, which was set at 100%. The values are presented as the means ± SEM, **p < 0.01 *versus* the control group and ^#^p < 0.05, ^##^*p* < 0.01*versus* the liver I/R group (*n* = 20).

**Figure 6:**
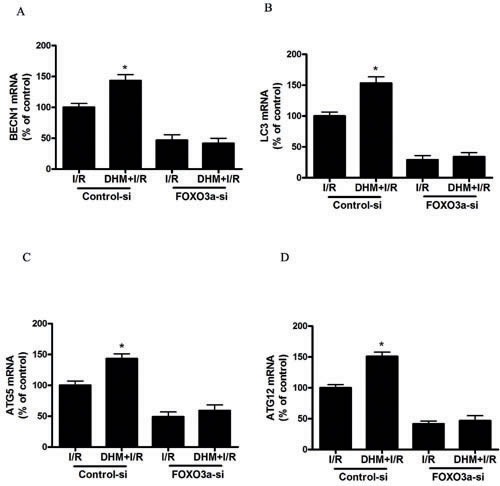
FOXO3a mediates DHM-induced autophagy-related genes After RNA interference of FOXO3a in mice, the mRNA level of **A.** BECN1, **B.** LC3, **C.** ATG5, and **D.** ATG12, was determined using RT-PCR as described previously. The results are expressed as a percentage of control, which was set at 100 %. The values are presented as the means± SEM, *p < 0.05, *versus* the liver I/R group (*n* = 20).

**Figure 7:**
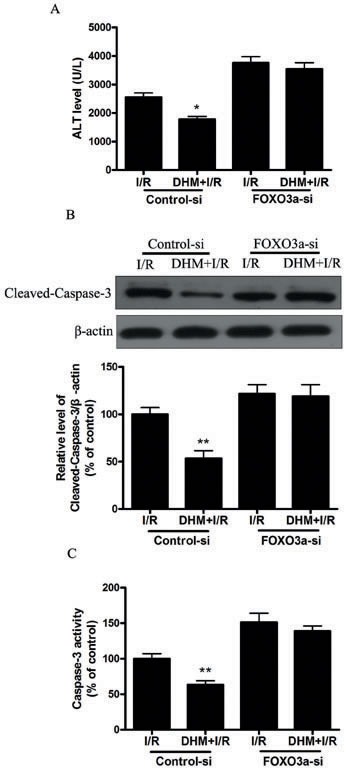
FOXO3a mediates the protective effects of DHM. After RNA interference of FOXO3a in mice **A.** ALT levels were determined using an ALT Determination Kit. **B.** Representative immunoblot of Cleaved-Caspase-3 protein levels in mice liver tissue. β-actin was the internal standard for protein loading. **C.** Caspase-3 activities in mice liver samples were determined using a Caspase-3 Activity Determination Kit. The results are expressed as a percentage of control, which was set at 100 %. The values are presented as the means± SEM, *p < 0.05, **p < 0.01 *versus* the liver I/R group (*n* = 20).

Because FOXO3a plays a critical role in autophagy, apoptosis, and stress resistance in various cell types [19], we sought to investigate the effect of DHM on FOXO3a. As shown in Figure 5A and 5B, liver I/R treatment resulted in a significant decrease in FOXO3a mRNA and protein levels. Notably, DHM pretreatment resulted in a significant increase in FOXO3a expression. Nuclear (i.e., transcription-dependent) and cytoplasmic (i.e., transcription-independent) functions were described for FOXO3a in autophagy promotion [20]. Nuclear translocation of FOXO3a stimulates autophagy in various cell types *via* the increased expression of numerous autophagy-related genes, including proteins in the autophagy core machinery. Interestingly, the data obtained by western blot of the subcellular fraction revealed that DHM administration significantly increased FOXO3a nuclear translocation (Figure 5C). It is now evident that the biological activity of FOXO3a is regulated predominantly by post-translational modifications, including phosphorylation and acetylation [21]. Figure 5D shows that DHM significantly elevated Ser588 phosphorylation of FOXO3a protein, but no changes in the Ser7, Ser253, Ser294, and Thr32 phosphorylation of FOXO3a were observed. Moreover, liver I/R treatment resulted in a significant increase in ac-FOXO3a, whereas DHM failed to restore ac-FOXO3a levels (Figure 5E). These data indicated that a phosphorylation modification of FOXO3a was required for DHM to trigger autophagy. To further determine whether FOXO3a could mediate DHM-induced autophagy, FOXO3a was inhibited with FOXO3a-specific siRNA prior to DHM in C57BL/6 mice. Notably, the inhibition of FOXO3a activity using FOXO3a-specific siRNA decreased DHM-induced autophagy-related genes (Figure 6A-6D). Additionally, the inhibition of FOXO3a activity attenuated the protective effects of DHM(Figure 7A-7C).

## DISCUSSION

To the best of our knowledge, this study provides the first evidence that (1) DHM reduces liver I/R injury in mice models; (2) the protective effect of DHM is mediated *via* induction of autophagy; and (3) DHM -induced autophagy is dependent on AMPK/FOXO3a signaling pathway.

*Ampelopsis grossedentata* is a medicinal and edible plant that is widely distributed in southern China. Its tender stem and leaves have been consumed as a health tea (Tengcha) for the prevention and treatment of the common cold, sore throat, and icteric viral hepatitis for hundreds of years [22]. DHM is a flavonoid compound found in *Hovenia dulcis*, in A. *grossedentata*, and in teas [23]. DHM possesses biological and pharmacological properties, including anti-oxidative, anti-inflammatory, and anti-cancer effects [10-12]. However, the effects of DHM on liver I/R injury have not been investigated. We showed that DHM treatment significantly improved the liver function of *in vivo* models. Our results are in line with those of previous studies evaluating the effects of DHM on D-galactosamine (GalN) or carbon tetrachloride induced liver injury [24, 25], and suggest that DHM could be developed as a novel therapeutic agent for the prevention and treatment of liver I/R injury.

Autophagy is a lysosome dependent mechanism by which dysfunctional or damaged intracellular organelles are broken down and recycled through the lysosomes. As a putative adaptive catabolic process, autophagy plays an important role in many human diseases such as ischemia and hypoxia [26, 27]. Autophagy is a dynamic process. We used several approaches to investigate whether I/R influences autophagic flux in the liver. We first examined the change of SQSTM1/p62 protein levels. This protein is selectively incorporated into autophagosomes through direct binding to LC3 and is efficiently degraded by autophagy [28]. We observed an evident decrease in SQSTM1 protein levels in the liver that were treated with I/R. Second, we used chloroquine (CQ), which is an inhibitor of the lysosomes and which causes an accumulation of autophagosomes due to a defect in the fusion between autophagosomes and lysosomes [29]. Western blotting revealed that CQ significantly increased the percentage of SQSTM1 protein abundance (Figure S1). These results confirmed intact autophagic flux in the I/R-treated liver. Inhibition of autophagy in these conditions can lead to increased cell death. Conversely, induction of autophagy can protect animals from liver I/R injury. Previous studies have documented that restoration or enhancement of autophagy may be a novel therapeutic modality to ameliorate liver function after I/R [30]. In contrast, suppression of I/R-induced autophagy by 3-MA or chloroquine worsened liver injury [31, 32]. These data strongly support the view that autophagy is a protective mechanism in I/R injury, and modulation of this process is a viable and novel therapeutic strategy for liver I/R injury. In our study, we demonstrated that DHM treatment could induce autophagy activity by enhancing autophagy-related gene expression during liver I/R. Our results also showed that the protective role of DHM may be partially due to autophagy induction. This finding is confirmed by data from *in vivo* studies, showing that DHM prevented hepatocytes from I/R insult and the beneficial effect was abrogated by treatment with WM.

We also examined the potential mechanisms of DHM-induced autophagy. The FOXO family has been shown to regulate autophagy in various systems, including skeletal muscle, cardiomyocytes, kidney, and liver. FOXO3a is likely involved in autophagy. For example, acute ethanol treatment increases the expression of autophagy related genes by activating FOXO3a in mouse liver and in primary cultured mouse hepatocytes [33]. Conversely, knockdown of FOXO3a decreased autophagosome foci in adult skeletal muscles [34]. FOXO3a is generally localized in the cell cytoplasm. However, post-translational modifications alter cellular localization from the cytoplasm to the nucleus, which induces the expression of autophagy-related genes, including ULK1/ATG1, PIK3C3/VPS34, BECN1/VPS30/ATG6, Atg4B/ATG4, LC3/ATG8, GabarapL1/ATG8, Atg12/ATG12, and Bnip3 [16, 35]. Our study results showed that DHM significantly increased FOXO3a expression, and enhanced FOXO3a nuclear translocation. In addition, DHM-induced autophagy and its beneficial effect were diminished by FOXO3a siRNA. These results indicated that DHM-induced autophagy *via* FOXO3a signaling would be necessary, and that FOXO3a-mediated autophagy plays an important role in the protective effect of DHM on liver I/R injury.

**Figure 8:**
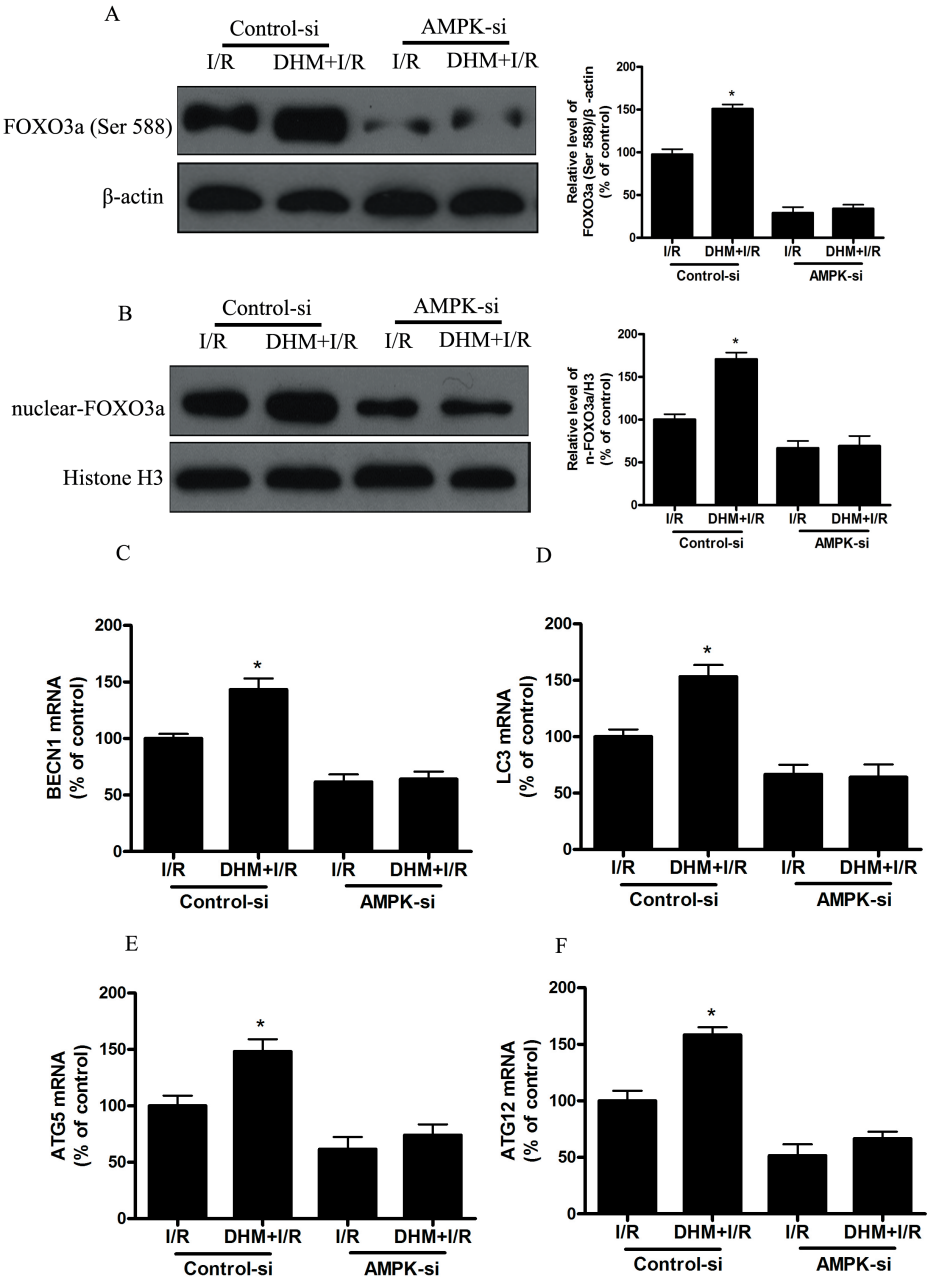
AMPK is involved in DHM-induced FOXO3a -dependent autophagy Mice were pretreated with AMPK-si prior to DHM treatment. **A.** p-FOXO3a(Ser588), protein expression levels were measured using Western blot analysis as described previously, with β-actin as an internal loading control. **B.** Representative immunoblot of nuclear FOXO3a levels, with H3 as an internal loading control. The mRNA level of **C.** BECN1, **D.** LC3, **E.** ATG5, and **F.** ATG12, was determined using RT-PCR as described previously. The results are expressed as a percentage of control, which was set at 100 %. The values are presented as the means± SEM, *p < 0.05 *versus* the liver I/R group (*n* = 20).

Phosphorylation and acetylation primarily regulate FOXO3a function and activity. The phosphorylation-dependent nuclear-cytoplasmic shuttling of FOXO3a involves a complex mechanism involving several upstream proteins, and one of the pathways involves AMPK activation. Previous studies demonstrated that AMPK enhanced the transcriptional activity of FOXO3a *via* direct Ser588 phosphorylation of FOXO3a [36, 37]. In study, we found that DHM treatment increased FOXO3a Ser588 and AMPK Thr127 expression. Moreover, AMPK gene silencing significantly suppressed I/R induced Ser588 phosphorylation of FOXO3a and nuclear translocation of FOXO3a, decreased DHM-induced autophagy-related genes (Figure 8). These findings suggest that the AMPK signaling pathway mediated DHM -induced nuclear translocation of FOXO3a. FOXO3a acetylation levels are regulated through acetylation by acetyltransferases (e.g., p300/CBP) and through deacetylation by SIRT [38, 39]. Localization of FOXO3a is determined by post-translational modifications. Of the various post-translational modifications, acetylation/deacetylation status is critical to determine the localization of FOXO3a. Acetylation causes export of FOXO3a to the cytosol, while deacetylation status of FOXO3a causes localization in the nucleus and induces transcription of target genes [40]. Consistent with previous studies, I/R significantly increased expression of acetylation-FOXO3a, which restrain FoxO3a in the cytosol in our study. However, we detected no significant changes in acetylated FOXO3a after DHM-pretreatment in the experimental model and speculate that FOXO3a acetylation does not play a critical role in DHM -induced autophagy.

In summary, our study clearly demonstrated that DHM could ameliorate liver I/R injury *via* induction of FOXO3a-mediated autophagy. Taken together, our data suggest that FOXO3a may be a therapeutic target for the development of novel treatments to prevent liver damage in patients with liver I/R.

## MATERIALS AND METHODS

### Hepatic ischemia procedure

Two-month-old C57BL/6 mice were maintained on a 12:12 hour light-dark phase and fed ad libitum spending 2 weeks adapting to these above conditions before the experiments. Under ketamine (60 mg/kg, intraperitoneal [i.p.]) and xylazine (8 mg/kg, i.p.) anesthesia, the liver hilum was exposed and the portal structures to the left and median lobes were occluded. The right lobes remained perfused to prevent intestinal congestion. After 60 minutes of ischemia, the clip around the left branches of the portal vein was removed to allow reperfusion. The sham control mice were prepared in a similar manner but a clip was not placed on the vasculature leading to the median and left lobes. After 5 hours of reperfusion, the mice were killed and the blood and ischemic liver tissue were collected [41]. The animal experiments were approved by Fuzhou General Hospital for Accreditation of Laboratory Animal Care.

### Drug treatment

DHM (Chengdu MUST Bio-Technology Co. Ltd, China, HPLC > 98 %) was dissolved in distilled water and administered daily by gavage at a dose of 100 mg/kg [42]. After DHM feeding for 7 days, the animals were randomly assigned to one of four groups as follows: (1) vehicle-treated sham (sham), (2) DHM-treated sham (DHM), (3) vehicle-treated ischemic (I/R), or (4) DHM-treated ischemic (DHM + I/R). Chloroquine (CQ) (Sigma-Aldrich, USA) dissolved in ddH_2_O and diluted with PBS to a final concentration of 60 mg/kg were intraperitoneally injected 30 min prior to the DHM treatment.

### Serum enzyme activity assays

Biochemical evaluation of liver injury was performed by quantifying alanine aminotransferase (ALT) serum activities using ALT test kits (SEA207Mu, USCN, China) according to the manufacturer's instructions [43].

### Caspase-3 activity assay

Caspase-3 activity was determined using a colorimetric assay based on the ability of caspase-3 to change acetyl-Asp-Glu-Val-Asp p-nitroanilide (Ac-DEVD-pNA) into a yellow formazan product (pNA). An increase in the absorbance at 405 nm was used to quantify the activation of caspase-3 activity. The liver tissues were rinsed with cold PBS and then lysed with lysis buffer for 15 minutes on ice. The lysates were centrifuged at 20,000 g for 15 minutes at 4 °C. Caspase-3 activity in the supernatant was assayed using the kit (Beyotime, China). Caspase-3 activity was expressed as a percentage of the enzyme activity compared with that of the control. Liver enzyme activities were normalized according to hepatic protein [44].

### RNA isolation and RT-PCR analysis

Total cellular RNA from liver tissues was isolated with RNAiso Plus (TaKaRa) and 1 μg of total RNA was reverse transcribed in a 20 μL reaction mixture using PrimeScript™ RT reagent Kit with gDNA Eraser (Perfect Real Time, TaKaRa) for cDNA synthesis (Invitrogen) according to the manufacturer's protocols. cDNA copy number was analyzed using the iQ5 Real-Time PCR Detection System (Bio-Rad) with the NovoStart^®^ SYBR qPCR Supermix (E090-01A, novoprotein). Gene expression was calculated using relative quantification normalized to the β-actin reference gene [45, 46]. Table 1 lists the primer sequences used for the amplification of target mouse genes. All primer sequences were checked with Genbank to avoid inadvertent sequence homology.

**Table 1:** Sequences of mouse primers used in quantitative RT-PCR

Target gene	Primer	Nucleotide sequence
FOXO3a	F R	5′-GGGGAACCTGTCCTATGCC-3′ 5′-TCATTCTGAACGCGCATGAAG-3′
BECN1	F R	5′-ATGGAGGGGTCTAAGGCGTC-3′ 5-TGGGCTGTGGTAAGTAATGGA-3′
ATG5	F R	5′-TGTGCTTCGAGATGTGTGGTT-3′ 5′-ACCAACGTCAAATAGCTGACTC-3′
ATG12	F R	5′-CGGAAGATTCAGAGGTTGTGCT-3′ 5′-CAGCCTTCAGCAGGATGTCAA-3′
LC3	F R	5′-TTATAGAGCGATACAAGGGGGAG-3′ 5′-CGCCGTCTGATTATCTTGATGAG-3′
β-actin	F R	5′-CGTGCGTGACATCAAAGAGAAG-3′ 5′-CAAGAAGGAAGGCTGGAAAAGA-3′

### Western blot analysis

The liver tissues were centrifuged for 15 minutes at 12,000 g and the resulting supernatant was transferred to a new tube. The protein concentrations were determined using a Bradford protein assay kit (Beyotime Company, Shanghai, China). The protein samples were separated by SDS-PAGE. Following protein transfer to PVDF membranes, the membranes were blocked and then incubated overnight at 4°C with antibodies and incubated with horseradish peroxidase-conjugated secondary antibodies for 1 hour. Protein signals were visualized using ECL detection system (Thermo Scientific) [47]. Table 2 provides further details and the identity and specificity of the primary antibodies.

**Table 2:** Antibodies used for the western blot experiments

β-actin	A5441	Sigma
FOXO3a	12829	Cell Signaling Technology
p-FOXO3a(Ser253)	9466	Cell Signaling Technology
p-FOXO3a(Ser7)	14724	Cell Signaling Technology
p-FOXO3a(Ser294)	5538	Cell Signaling Technology
p-FOXO3a(Ser588)	N/A	Made by Abmart
p-FOXO3a(Thr32)	9464	Cell Signaling Technology
Cleaved-Caspase-3	9664	Cell Signaling Technology
SQSTM1/P62	Ab56416	Abcam
AMPK	5831	Cell Signaling Technology
p-AMPK(Thr172)	2535	Cell Signaling Technology
anti-mouse (secondary antibody)	A0208	Beyotime Company
anti-rabbit (secondary antibody)	A0216	Beyotime Company
anti-goat (secondary antibody)	A0181	Beyotime Company
H3	SAB4500352	Sigma
Ac-lysine	9814	Cell Signaling Technology

### Nuclear-cytoplasmic fractionation

Nuclear-cytoplasmic fractionation was conducted using NE-PER™ Nuclear and Cytoplasmic Extraction Reagents (Life Technologies, 78833) according to the manufacturer's instructions. Protein concentration was determined using BCA protein assay reagent (Beyotime, P0010) with bovine serum albumin as the standard, and equal amounts of each cell lysate were separated by 12% SDS-PAGE. To assess the purity of fractionation, cytoplasmic, and nuclear fractions were confirmed by immunoblotting using anti-Actin (1:5000, Sigma, A5441) as a cytoplasmic marker, and anti-Histone H3(1:1000, Sigma, SAB4500352) as a nuclear marker, respectively.

### Immunoprecipitation

The liver tissues were lysed with cell lysis buffer (Beyotime Company, P0013). Lysates were clarified by centrifugation at 12,000 g for 15 min and were used for immunoprecipitation. A total of 2 μg of antibody was incubated with 500-1000 μg of protein overnight at 4°C. Next, protein A beads (Beyotime Company, P2006) were added and the mixture was incubated overnight at 4°C. After incubation, the beads were washed 3 times, solubilized in 40 μl 3xSDS sample buffer (Cell Signaling Technology, 7722), and analyzed by western blotting.

### RNA interference of FOXO3a in mice

To evaluate the effects of FOXO3a, Atg5 and AMPK interference *in vivo*, FOXO3a-siRNA, Atg5-siRNA or AMPK-siRNA (Invitrogen) hydrodynamic injection was given to mice. Briefly, 200 nmol/kg siRNA was diluted in physiological saline and then injected into the tail vein within 5-10 seconds. FOXO3a-specific siRNA, Atg5-siRNA or AMPK-siRNA was given to mice 2 hours prior to DHM expoure to inhibit FOXO3a, Atg5 or AMPK. Scrambled siRNA was used as a control [9]. The knockdown efficiency is included in Figure S2.

### Statistical analysis

Data were analyzed using GraphPad Prism-5 software. All experimental data are expressed as the mean ± SEM, and One-way ANOVA was used to determine statistical significance, and *P* < 0.05 was considered to be statistically significant.

## SUPPLEMENTARY MATERIALS FIGURES


