# Supplementary material for: Dihydromyricetin protects against liver ischemia/reperfusion induced apoptosis via activation of FOXO3a-mediated autophagy
Source: Oncotarget. 2016 Oct 25;7(47):76508–22. doi: 10.18632/oncotarget.12894 (PMC5363527; doi:10.18632/oncotarget.12894)
Supplement: Supplementary file 1 [file oncotarget-07-76508-s001.pdf]

Dihydromyricetin protects against liver ischemia/reperfusion induced apoptosis *via* activation of FOXO3a-mediated autophagy

Supplementary Material

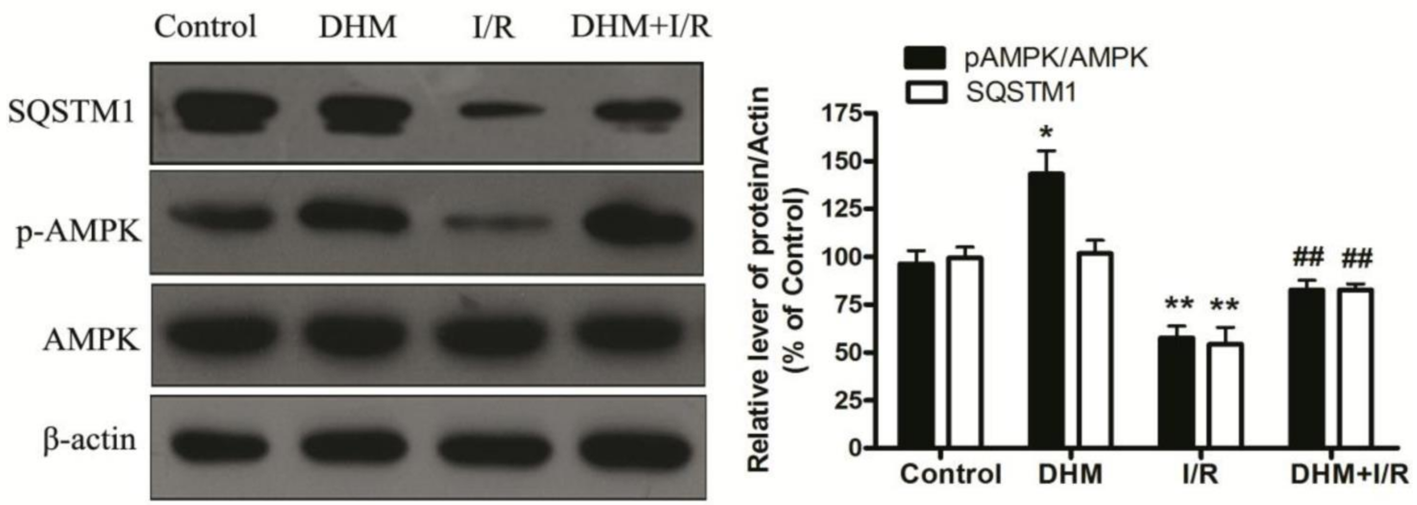

Fig. S1 Effects of DHM on the expression of SQSTM1 and AMPK suppressed by liver I/R injury in C57BL/6 mice. The protein level of (A)SQSTM1, (B)p-AMPK, was determined using wstern-blot as described previously. The results are expressed as a percentage of control, which was set at 100 %. The values are presented as the means ± SEM, *p < 0.05, **p < 0.01 versus the control group, and ##p < 0.01 versus the liver I/R group(n=20).

[illegible]

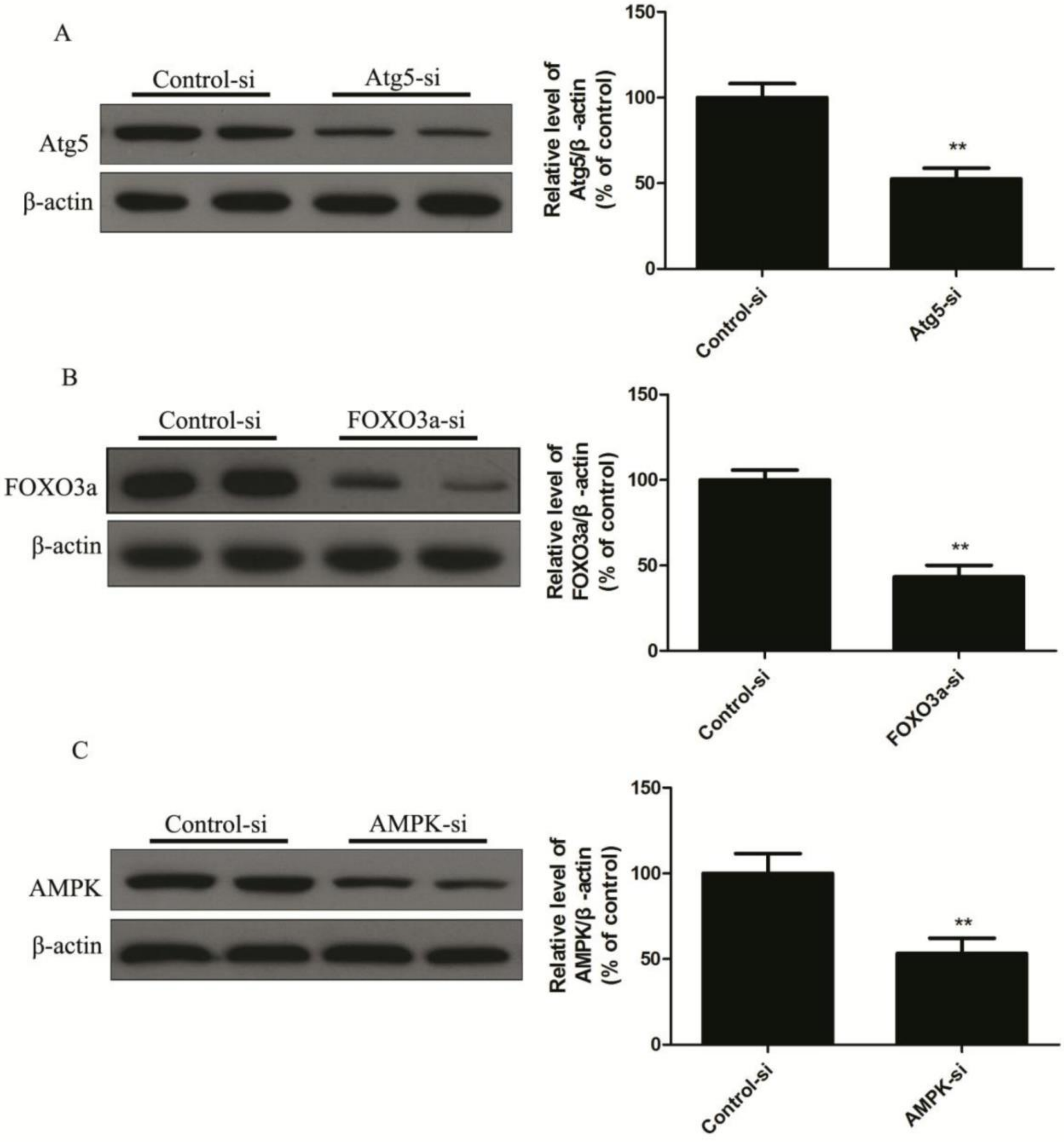

Fig. S2 The knockdown efficiency of si-RNA approaches is included in our study. The protein level of (A)Atg5, (B)AMPK, (C)FOXO3a, was determined using western-blot as described previously. The results are expressed as a percentage of control, which was set at 100 %. The values are presented as the means ± SEM, **$p < 0.01$ versus the control group (n=20).
